# Exploring time-killing and biofilm inhibition potential of bioactive proteins extracted from two varieties of *Pleurotus ostreatus*

**DOI:** 10.3389/fmicb.2024.1456358

**Published:** 2024-11-12

**Authors:** Reena Gangwar, Mohamed M. Salem, Vineet Kumar Maurya, Mounir M. Bekhit, Nisha Singh, Amro Abd Al Fattah Amara, Ram Kumar Sahu, Mohamed A. Ibrahim

**Affiliations:** ^1^Department of Botany and Microbiology, Hemvati Nandan Bahuguna Garhwal University (A Central University), Srinagar Garhwal, India; ^2^College of Medicine, Huazhong University of Science and Technology, Wuhan, China; ^3^Department of Pharmaceutics, College of Pharmacy, King Saud University, Riyadh, Saudi Arabia; ^4^Department of Biochemistry, Hemvati Nandan Bahuguna Garhwal University (A Central University), Srinagar Garhwal, India; ^5^Department of Biochemistry, Faculty of Science, University of Lucknow, Lucknow, India; ^6^Department of Protein Research, Genetic Engineering and Biotechnology Research Institute, City of Scientific Research and Technological Applications, Universities and Research Centre District, New Borg El-Arab, Egypt; ^7^Department of Pharmaceutical Sciences, Hemvati Nandan Bahuguna Garhwal University (A Central University), Chauras Campus, Tehri Garhwal, India

**Keywords:** bioactive peptides, *Pleurotus ostreatus*, time-kill, antihemolytic, antimicrobial, antibiofilm, MRSA, nystatin resistant

## Abstract

**Introduction:**

Dental caries, caused by oral microbial pathogens, are a global health concern, further exacerbated by the presence of methicillin-resistant *Staphylococcus aureus* (MRSA). Bioactive proteins and peptides (BAPs) exhibit potent antimicrobial properties, targeting multiple cellular mechanisms within pathogens, reducing the likelihood of resistance development. Given the antimicrobial potential of BAPs, this study aimed to compare the efficacy of BAPs extracted from cultivated (*Pleurotus ostreatus*, PoC) and wild (*Pleurotus ostreatus*, PoW) mushrooms against pathogens responsible for dental caries.

**Methods:**

BAPs were extracted from both PoC and PoW using a TCA-acetone method. Antimicrobial activities were tested against seven bacteria and one fungus using agar well diffusion and MIC determination. Antibiofilm activity was assessed via modified CV assay, while DPPH and erythrocyte lysis tests evaluated free radical scavenging.

**Results:**

PoC showed superior antimicrobial efficacy, with lower MIC and MBC values, and disrupted biofilm integrity at increasing concentrations. PoW exhibited better antioxidant activity with higher DPPH scavenging, though its antimicrobial efficacy was slightly lower than PoC.

**Discussion:**

Both PoC and PoW BAPs inhibited dental pathogens, with PoC showing stronger inhibition against MRSA and nystatin-resistant Candida albicans. This suggests BAPs may target additional cellular mechanisms beyond membranes, PBPs, and ergosterols. Despite PoW’s stronger antioxidant properties, both BAPs had comparable antibiofilm activity. These findings suggest complementary actions of BAPs from PoC and PoW both, in treating dental caries, offering broad-spectrum antimicrobial and antioxidant benefits.

## 1 Introduction

Dental caries is a highly widespread chronic ailment that is observed on a global scale. People are vulnerable to this illness at all stages of their lifespan. Globally, approximate 2 billion adults and 514 million children suffer from dental caries. In India, overall 54.16% population is affected with dental caries, while 52% are affected in the age group of 3–18 years. High sugar intake, inadequate salivary flow, insufficient fluoride exposure, enamel defects, poor oral hygiene, and inappropriate methods of feeding infants are non-microbial causes of dental caries (Selwitz et al., 2007). Meanwhile, microbial causes involve a complex interaction of oral bacteria with fermentable carbohydrates, which produce organic acids and damage the entire tooth, ultimately leading to dental caries.

Among the oral microbiota of humans, seven bacterial pathogens, involved in dental problems were selected in the present study. *Streptococcus mutans, Streptococcus oralis* (Banas et al., 2016), *Lactobacillus* spp., and *Actinomyces* spp. are involved in the initiation and progression of dental caries (Loesche, 1986; Nyvad and Takahashi, 2020), while *Enterococcus faecalis* is a commonly-isolated species from persistent apical periodontitis (Rocas et al., 2004). Common nosocomial bacteria *Staphylococcus aureus* is believed to be associated with peri-implantitis (Persson and Renvert, 2013), and therapy-resistant cases of periodontitis (Fine, 1994) *Escherechia coli* was added to the study because it is a common bacteria of human microbiome which sometime becomes opportunistic pathogen. *Candida albicans* causes oral thrush in persons with weak immunity (Patel, 2022).

Presence of drug resistance in *S. aureus* is another serious issues in handling dental problems. Antimicrobial peptides (AMPs) constitute a class of therapeutic molecules, which are produced by fungi, plants, invertebrates and vertebrates as their non-specific host defence system against the microbial pathogens (Akbarian et al., 2022; Browne et al., 2020). AMPs exhibit significant structural diversity and primarily target the cell membranes of pathogens, displaying a lower propensity for the development of resistance. Currently, 885 protein based compounds are under clinical studies, indicating high market potential for protein and peptides based therapies.

AMPs exhibit a range of desirable properties, including cidal or static activity against drug-resistant microorganisms, immunomodulatory effects, antioxidant capabilities, wound healing promotion, antibiofilm activity, and anticancer properties. Among these, their effectiveness against drug-resistant microbes is particularly significant in the current context. Given their proteinaceous nature, the proteins extracted from natural sources can be explored to confirm the presence of AMPs within them. Therefore, protein extraction becomes crucial steps in the discovery of AMPs. Among the widely used protein extraction protocols [Tri-Chloro-Acetic acid (TCA), TCA-Acetone, Ammonium sulfate and Tris-phenols based protocols] (Isaacson et al., 2006; Novak and Havlicek, 2016). TCA-Acetone based methods was selected in the present work. TCA-acetone method is robust, less instrument intensive and provides good quality of protein precipitate which is free from contamination and easy to solubilize for further experiments (Niu et al., 2018).

In present work, mushrooms were chosen as source of proteins for AMPs isolation, for two reasons. Firstly, mushrooms are rich in proteins and secondly, they are relatively less explored for AMPs. In addition, mushrooms have proven nutraceutical and therapeutic benefits, and a plethora of bioactive metabolites such as polysaccharides, alkaloids, phenolics, sterols, fatty acids, proteins and peptides, etc., have been reported in mushrooms (Anusiya et al., 2021; Assemie and Abaya, 2022; Gangwar et al., 2023). Therapeutic potential of aqueous and organic solvent based extracts of mushrooms, such as Reishi (*Ganoderma lucidum*), Shiitake (*Lentinula edodes*), Turkey Tail (*Trametes versicolor*), Cordyceps (*Cordyceps sinensis*) etc., have been reported. Antimicrobial potentials of the proteins extracted from *G. lucidum* (Marimuthu and Mahendran, 2019), *Ganoderma resinaceum*, *Russula fragilis*, *Inocybe grammata* (Hearst et al., 2010) and *Agaricus bisporus* (Gangwar and Maurya, 2022) have also been evaluated by different research groups. But, there are scanty studies on therapeutic potential of *Pleurotus ostreatus* (wild and cultivated varieties) and in our knowledge any study focusing on AMPs from these mushrooms is lacking. It is anticipated that the antibacterial activity of total proteins will reflect the presence of AMPs in the proteins. Therefore, antimicrobial activity based screening of extracted proteins, referred as “Bioactive proteins” (BAPs) further, would be helpful in identifying mushrooms as the source of novel AMPs (Jakubczyk et al., 2020; Lavanya and Subhashini, 2013; Magana et al., 2020).

*Pleurotus ostreatus*, the “oyester mushroom” ranks among the top five cultivated mushrooms within the Pleurotaceae family, which comprise about 40 species including *P. eryngii, P. ostreatus, P. citrinopileatus, P. ferulae* etc. Beyond its recognized nutritional and culinary value, *P. ostreatus* holds medicinal significance. The *in-vitro* antitumour activities of ethanol extract, aqueous extracts and Selenium containing polysaccharide fraction (Se-POP-3) of *P. ostreatus* were reported in A549, SKOV3, HepG2, CaCO2, and MCF-7 cells lines, where *P. ostreatus* shown to inhibit metastasis, induce apoptosis, and enhance proapoptotic Bax proteins. Antibacterial activities against *E. coli, S. aureus, Micrococcus luteus, Proteus vulgaris, Pseudomonas aeruginosa*, and *Proteus mirabilis,* as well as antifungal activities against *C. albicans, Fusarium* sp., and *Rhizoctonia solani*, along with immunomodulatory and antioxidant properties of *P. ostreatus* have also been documented (Goswami et al., 2021; Nguyen et al., 2016; Waktola and Temesgen, 2020).

AMPs are also being explored as potential cure against the problem of drug resistant. Therefore, in present study seven antibiotics (six antibacterial and one antifungal) were used to compare the BAPs with standard antimicrobials. Six antibiotics used in the study were selected on the base of their mode of action, i.e., targeting bacterial cell wall synthesis or bacterial protein synthesis, and the range of spectrum, i.e., broad spectrum or narrow range. Amoxicillin and Ampicillin are broad spectrum, cell wall synthesis inhibiting antibiotics, while methicillin is effective against Gram positive bacteria only. Among protein synthesis inhibiting antibiotics, azithromycin is a broad range antibiotic with more efficacy against Gram negative bacteria, erythromycin is active against Gram positive, and clindamycin are active against Gram positive anaerobic bacteria. Nystatin was used due to it antifungal properties.

**Figure 1:**
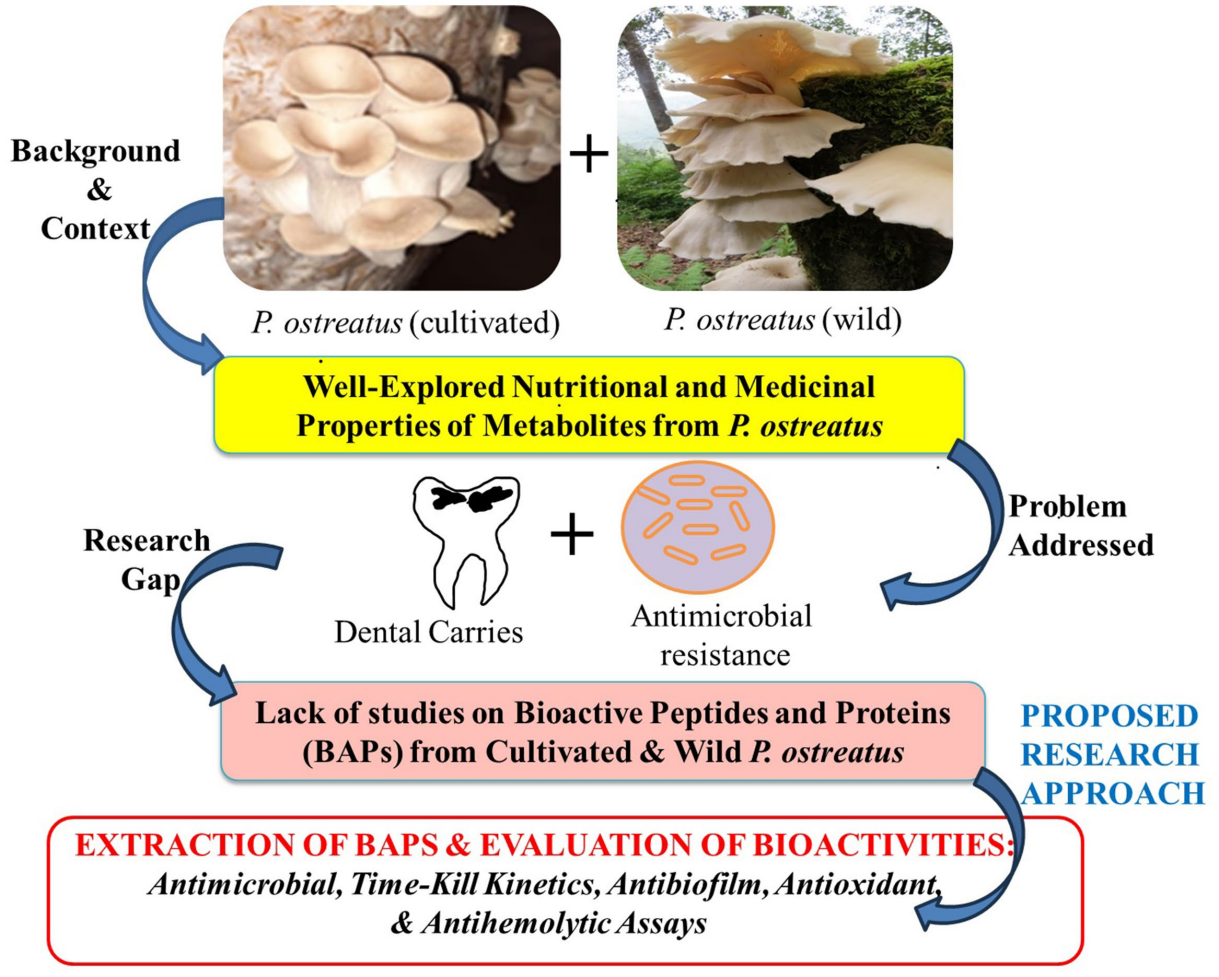
Rationale of the work performed in this study.

While the medicinal benefits of the PoC have been extensively documented, the potential therapeutic properties of its wild counterpart, i.e., PoW remains largely unexplored. Previous research has mainly focused on ethanol and aqueous extracts, as well as polysaccharide fractions, with little attention given to “Bioactive proteins” (BAPs) or “antimicrobial peptides” (AMPs). Furthermore, there is a dearth of information regarding the comparison between wild and cultivated varieties of *P. ostreatus,* in terms of their antimicrobial proteins and peptides. Consequently, our current study endeavors to unveil the medicinal attributes, including antimicrobial, antibiofilm, antihemolytic, and antioxidant properties, of the BAPs extracted from both the wild and cultivated varieties of *P. ostreatus*, along with a comprehensive comparison between the two. The rationale of the present study has been graphically represented in the Figure 1.

## 2 Materials and methods

### 2.1 Identification of mushrooms

Fresh fruiting bodies (young to mature basidiocarps) of *P. ostreatus*, wild and cultivated varieties (PoW and PoC), were collected. PoW samples were collected from the forest of Pedkhal block, Pauri Garhwal, and the PoC samples were collected from local market of Srinagar Garhwal. The collected specimens were processed following the standard procedures of sample drying and storage. Collected mushrooms were identified based on their Macro and microscopic characters (Gangwar et al., 2023; Ghosh et al., 2020). The compound microscope (Olympus CH20i) was used for microscopic observations and images were captured using Olympus CX21iLED camera mounted to it. Samples were dried and stored for further experiments. Samples were dried in wooden drier, equipped with blower for circulation of warm air. The temperature of 40–45°C was maintained during sample drying.

### 2.2 Extraction of total soluble proteins

One gram of dried mushroom samples was ground into fine powder in presence of liquid nitrogen using pre-chilled mortar-pestle. Trichloroacetic acid (TCA)-Acetone precipitation method was used for extraction of total soluble proteins (Niu et al., 2018) with slight modification. Briefly, fine grinded mushrooms were suspended into chilled “protein extraction buffer” (0.015 M Tris, 0.25 M EDTA, and 0.5 mL of *β*-mercaptoethanol in 100 mL of triple distilled water, with final pH-7.2), suspension was centrifuges at 4,500 rpm for 20 min, and the supernatant was isolated. Proteins were precipitated by adding 10% (v/v) TCA-acetone to the supernatant in gradual manner, followed by overnight incubation at 4°C. Following the period of incubation, the precipitate was recovered using centrifugation at a speed of 12,000 × g for 15 min at a temperature of 4°C. The supernatant was discarded, and the protein pellet was washed three times using cooled acetone in order to eliminate any remaining TCA residues. The pellet was air stored in deep freezer for further experiment.

Protein concentration was measured using Modified Lowry’s Method, taking 1 mg/mL Bovine serum albumin (BSA) as the standard.

### 2.3 Antibacterial assays

#### 2.3.1 Microorganisms

The seven species of oral and perioral bacteria; *S. oralis* (MTCC-2696)*, L. acidophilus* (MTCC-10307)*, S. mutans* (MTCC-497)*, A. viscosus* (MTCC-22)*, S. aureus* (MTCC-96)*, E. coli* (MTCC-68)*, E. faecalis* (MTCC-439); and one fungus *C. albicans* (MTCC-183) was obtained from the Microbial Type Culture Collection and Gene Bank (MTCC) located at the Institute of Microbial Technology in Chandigarh, India (Table 1) and maintained in pure culture form.

**Table 1:** Details of the MTCC No., growth conditions, incubation time and antibiotic resistant status of the microorganism used in the study.

Microorganisms (Gram +/−)	MTCC No.	Incubation period	Incubation temperature	Antibiotic resistance status
*S. mutans* (+)	MTCC-497	24 h	37°C	Susceptible*
*S. oralis* (+)	MTCC-2696	24 h	37°C	Susceptible*
*L. acidophilus* (+)	MTCC-10307	48 h	37°C	Susceptible*
*A. viscosus* (+)	MTCC-22	24 h	37°C	Susceptible*
*S. aureus* (+)	MTCC-96	24 h	37°C	Methicillin resistant
*E. coli* (−)	MTCC-68	24 h	37°C	Susceptible*
*E. faecalis* (+)	MTCC-439	24 h	37°C	Susceptible*
*C. albicans*	MTCC-183	48 h	25°C	Nystatin resistant

*Susceptible to the six antibiotics used in this study.

Wherever mentioned, the Nutrient agar/broth or Muller Hinton Agar (MHA) (Hi-Media) was used for bacterial growth, while Potato Dextrose Agar (PDA)(Hi-Media) was used for the growth of *C. albicans*. The bacterial and fungal pathogens were grown under aerobic conditions, at incubation temperature of 37°C and 25°C, respectively. Two of the pathogens, *A. viscosus* and *L. acidophilus* were facultative anaerobe and microaerophilic, respectively. Under the aerobic growth conditions used in present work, the growth of both the bacteria, especially *L. acidophilus* was slower, and took extra incubation time (upto 48 h) for proper growth, compared to other bacteria.

For antimicrobial assays, the O.D. of actively growing bacterial cultures was adjusted to 1.5 × 10^8^colony-forming units (CFU)/mL (equivalent to 0.5 McFarland standards).

#### 2.3.2 Antimicrobial activity of BAPs

Extracted total soluble proteins were solubilized in SDS-lysis buffer (SLB), and the stock of 300 μg/mL concentration was prepared for antibacterial activity experiments (Chandra et al., 2015). Total proteins also contain small peptides, and this solution was treated as BAPs for further experiments. 50–100 μL of this stock was poured into the wells, cut into MHA agar plates. SLB without any antimicrobial agent, and standard antibiotics were used as negative and postive controls, respectively.

#### 2.3.3 Standard antibiotics

Seven standard antibiotics namely Erythromycin, Azithromycin, Amoxicillin, Ampicillin, Clindamycin, methicillin and Nystatin were used to compare the antibacterial potential of BAPs with them. All antibiotic discs were purchased from Hi-Media.

#### 2.3.4 Agar well diffusion method

The diffusion technique using agar wells was utilized to ascertain the BAPs’ capacity to inhibit the growth of bacteria. For antibacterial activity 100 μL suspension of microbial pathogens (bacteria or fungal spores) having O.D. equivalent to 0.5 McFarland was spread uniformly on MHA plates. Further, 7.0 mm diameter wells were cut into agar plates pre-inoculated with bacteria, and 100 μL of solution containing the required amounts of BAPs (5 μg, 15 μg, or 30 μg) was added to each well. Plates were incubated at required temperature and the zone of inhibitions (ZOIs) that appeared around the wells was measured in millimeters after incubation. All the experiments were performed in triplicates and ZOIs were calculated by subtracting the well diameter from the diameter of the clear zone around the well (Chandra et al., 2015; Erhonyota et al., 2023).

### 2.4 Determination of MIC and MBC

#### 2.4.1 Minimal inhibitory concentration

To determine the MIC of BAPs against the tested microorganisms, the two-fold serial dilution approach was used. In this method 3.0 mL of sterile nutrient broth media was taken into test tubes and graded doses of BAPs, dissolved in equal volume of 100 μL were added. Each tube was further inoculated with 100 μL of bacterial or fungal culture (as prepared in section 2.3.1) to achieve final CFU count of 5 × 10^6^ CFU/mL. 2.0 μg/mL to 256 μg/mL concentrations of BAPs was used for MIC testing. The absorbance of MIC tubes was measured at 600 nm after incubation (Ibrahim and Al-Mizraqchi, 2024; Parvekar et al., 2020).

#### 2.4.2 Minimal bactericidal concentration

The MBC was found by sub-culturing all of the macroscopically clear tubes and placing the tube with the least amount of turbidity in the series adjacent to clear tubes on agar plates. This allowed for the determination of the MBC. Prior to sampling, the tubes were subjected to gentle mixing employing a sterilized micropipette, after which a 10 μL portion was taken from each tube. In accordance with the methodology outlined by Adeoye-Isijola et al. (2022), every aliquot was carefully applied onto an individual nutritional agar plate devoid of antibiotics. The application was performed by streaking a single line down the center of each plate. The samples were permitted to undergo absorption into the agar medium until the surface of the plate exhibited a desiccated appearance, which occurred after a duration of 30 min. The plates used for evaluating the MBC were subjected to incubation. Following the designated incubation durations, the MBC values for the BAPs were determined by identifying the lowest concentration of BAPs that exhibited no bacterial growth on the agar plates (Olajuyigbe and Afolayan, 2012).

### 2.5 Time kill assay

The antibacterial properties of BAPs from PoC and PoW were further analysed using time-kill assay (Wang et al., 2012). Briefly, the microbial suspensions were adjusted to 1 × 10^6^ CFU/mL (Ferro et al., 2015). BAPs from both the mushrooms were added to the suspension at concentrations 1X, 2X, 3X, and 4X MBC (Huang et al., 2011). The suspensions were incubated at desired temperatures with gentle agitation. 100 μL of aliquots were collected after every 0, 4, 8, 16, 20, and 24 and 48 h of incubation, from each tube and inoculated on agar plates, followed by CFU counting.

### 2.6 Assessment of biofilm inhibitory potential of BAPs

#### 2.6.1 Initial cell attachment inhibition

The effect of BAPs from PoC and PoW on biofilm formation was evaluated following the method of Jadhav et al. (2013) and Sandasi et al. (2010). Different concentrations (equivalent to 1X MIC, and 2X MIC) of the BAPs from PoC and PoW mushroom*s* were prepared. Individual sterile test tubes were each supplemented with 100 μL of every dilution. A negative control was established by adding an equivalent volume of distilled water. Subsequently, a volume of 100 μL of the bacterial cultures was introduced into the test tubes. The experiment was conducted in triplicate. In order to validate the sterility of the medium, the nutrient broth was included as an extra control. The tubes were hermetically sealed and placed in a sterile environment, where they were kept at a temperature of 37°C for 24 h. This incubation period facilitated the adherence of microbial cells, promoting the biofilm formation. The evaluation of biofilm formation was conducted through the utilization of the crystal violet assay, as stated in the subsequent sections.

#### 2.6.2 Biofilm inhibition

The impact of BAPs on the growth and development of biofilm was assessed following the methodology outlined by Djordjevic et al. (2002) and Jadhav et al. (2013), with certain adaptations. Biofilms were let to develop for 6 h before the introduction of BAPs obtained from PoC or PoW. Biofilm production was initiated by inoculating 100 μL of the bacterial culture (made according to the methodology outlined in Section 2.6.1) into sterile test tubes containing 5 mL of sterile broth, with each condition replicated three times. The tubes were thereafter covered and placed in an incubator at a temperature of 37°C for 6 h, with the purpose of facilitating the attachment of cells and the subsequent creation of a biofilm. Bacterial proliferation was assessed using the measurement of O.D. at a wavelength of 600 nm. After a period of incubation, 100 μL of each stock solution containing bioactive compounds from the collected mushroom samples was introduced into individual test tubes, resulting in a final volume of 2.0 mL for each test tube. Negative controls were prepared by adding equal amounts of SLB. Following the application of BAPs derived from collected samples, the pre-existing biofilms were subjected to a 24 h incubation period. After the incubation period, the biofilms were evaluated for biomass attachment using the crystal violet technique.

#### 2.6.3 Biofilm biomass screening

The modified crystal violet (CV) test, outlined by Djordjevic et al. (2002) and Jadhav et al. (2013), was utilized to investigate the indirect measurement of attachment of cells for all eight dental caries microorganisms. After the 24 h incubation period as described in section 2.6.1, and another 24 h incubation period as described in Section 2.6.2, the culture media was carefully aspirated from each well, and the test tubes were thereafter rinsed three times with sterile distilled water in order to eliminate any bacterial cells that were not firmly adhered. The test tubes were dried by allowing air to naturally evaporate the moisture, followed by further drying in an oven at a temperature of 60°C for a duration of 45 min. Subsequently, the cells within the biofilm were subjected to staining using a 0.1% solution of crystal violet (3 mL) and were subsequently incubated at ambient temperature for a duration of 15 min. Subsequently, the test tubes underwent a triple washing procedure employing sterile distilled water in order to eliminate any surplus discoloration. Subsequently, a volume of two milliliters of ethanol with a concentration of 95% was introduced into the test tubes in order to facilitate destaining. The absorbance of the resulting solution was subsequently determined at a wavelength of 595 nm using a spectrophotometer. The degree of biofilm inhibition was determined by comparing the quantity of biofilm formed in the presence of BAPs to the amount of biofilm formed in two reference conditions: the absence of BAPs (considered as 100% biofilm) and the control with sterile media (considered as 0% biofilm). The data obtained from a minimum of three independent biological replicates were subjected to averaging. The percentage inhibition of biomass production for each concentration of BAPs from collected mushroom samples was determined by utilizing the mean absorbance (O. D. 595 nm), as per the equation provided:


$$\mathrm{Percentage}\;\mathrm{inhibition}=100-\frac{\begin{array}{l} O.D.\left(595\;\mathrm{nm}\right)\;\mathrm{of}\;\mathrm{the}\;\mathrm{tubes}\;\\ \mathrm{treated}\;\mathrm{with}\;\mathrm{BAPs} \end{array}}{\begin{array}{l} O.D.\left(595\;\mathrm{nm}\right)\;\mathrm{of}\;\mathrm{Control}\;\\ \mathrm{tube}\;\mathrm{without}\;\mathrm{BAPs} \end{array}}\times 100$$

### 2.7 Antioxidant assays

#### 2.7.1 DPPH assay

Antioxidant potential of the BAPs from PoC and PoW was measured using DPPH assay. Varying concentration of extracted proteins (0.2–1 mg/mL) dissolved in 1.0 mL, was added to 2.0 mL of the DPPH solution, and tubes were incubated in dark. The absorbance was assessed at a wavelength of 517 nm. Positive and negative controls were employed, with ascorbic acid representing the positive control and a pure solvent mixed with DPPH serving as the negative control. IC-50 values were determined through graphical analysis (Gangwar et al., 2023).

#### 2.7.2 Antihemolytic assay

The assessment of the antihemolytic activity of the BAPs was conducted using the methodology given by Alinezhad et al. (2013) and Nabavi et al. (2010) with certain modifications. A sample of goat blood, measuring 10 mL, was obtained from a nearby slaughterhouse and stored in EDTA tubes with a concentration of 10%. Subsequently, the sample was subjected to centrifugation at a speed of 3,000 rpm for a duration of 10 min. The liquid portion above the sedimented erythrocytes was extracted, and the pellet containing the erythrocytes was subjected to three washes using a 0.2 M phosphate-buffered saline solution with a pH of 7.4, as described by Karim et al. (2020). The erythrocytes were then re-suspended in a saline solution containing 0.9% sodium chloride. A 4% (v/v) suspension of erythrocytes was prepared by suspending them in phosphate-buffered saline. Subsequently, a total of 0.5 mL of BAPs at four varying concentrations (100 μg/mL, 250 μg/mL, 500 μg/mL and 1,000 μg/mL) were introduced into a 2.0 mL suspension of erythrocytes. The resulting mixture was then diluted to a final volume of 5.0 mL using a saline buffer. The mixture was subjected to incubation for a duration of 5 min at ambient temperature, followed by the addition of 0.5 mL of hydrogen peroxide solution to initiate the process of oxidative destruction of the membrane lipids. The samples were once again subjected to incubation at a temperature of 37°C in a shaking incubator for a duration of 3 h. Following the incubation period, the samples were subsequently subjected to centrifugation at a speed of 3,000 rpm for a duration of 10 min. The resulting supernatant was then analyzed for absorbance at a wavelength of 540 nm. The concentration of hydrogen peroxide in the reaction mixture was manipulated in order to achieve complete hemolysis of RBCs within a time frame of 3 h. This particular tube, in which 100% hemolysis of RBCs was achieved, was designated as the negative control. The degree of hemolysis was evaluated relative to the complete hemolysis induced by hydrogen peroxide (H_2_O_2_), which served as the negative control. The standard substance utilized in this experimental study was L-ascorbic acid, with concentrations ranging from 100 to 1,000 μg/mL. The blank solution, serving as a control, was generated by combining 0.2 M PBS and 0.82 M H_2_O_2_.


$$\mathrm{Percentage}\;\mathrm{hemolysis}=\frac{\begin{array}{l} \mathrm{Absorbance}\;\left(540\;\mathrm{nm}\right)\;\mathrm{of}\;\mathrm{sample}-\\ \mathrm{Absorbance}\;\left(540\;\mathrm{nm}\right)\;\mathrm{of}\;\mathrm{blank} \end{array}}{\begin{array}{l} \mathrm{Absorbance}\;\left(540\;\mathrm{nm}\right)\;\mathrm{of}\;\mathrm{negative}-\\ \mathrm{Absorbance}\;\left(540\;\mathrm{nm}\right)\;\mathrm{of}\;\mathrm{blank} \end{array}}\times 100$$

%inhibitionofhemolysis=100−%Hemolysis

### 2.8 Statistical analysis

Tuckey-test and one way ANOVA test were performed using SPSS 16.0. A *p*-value of less than 0.05 was considered as statistically significant. The experiments were conducted in triplicate, and the outcomes were determined by calculating the mean value along with its corresponding standard deviation (M ± S.D.).

## 3 Results

### 3.1 Identification of mushrooms

**Figure 2:**
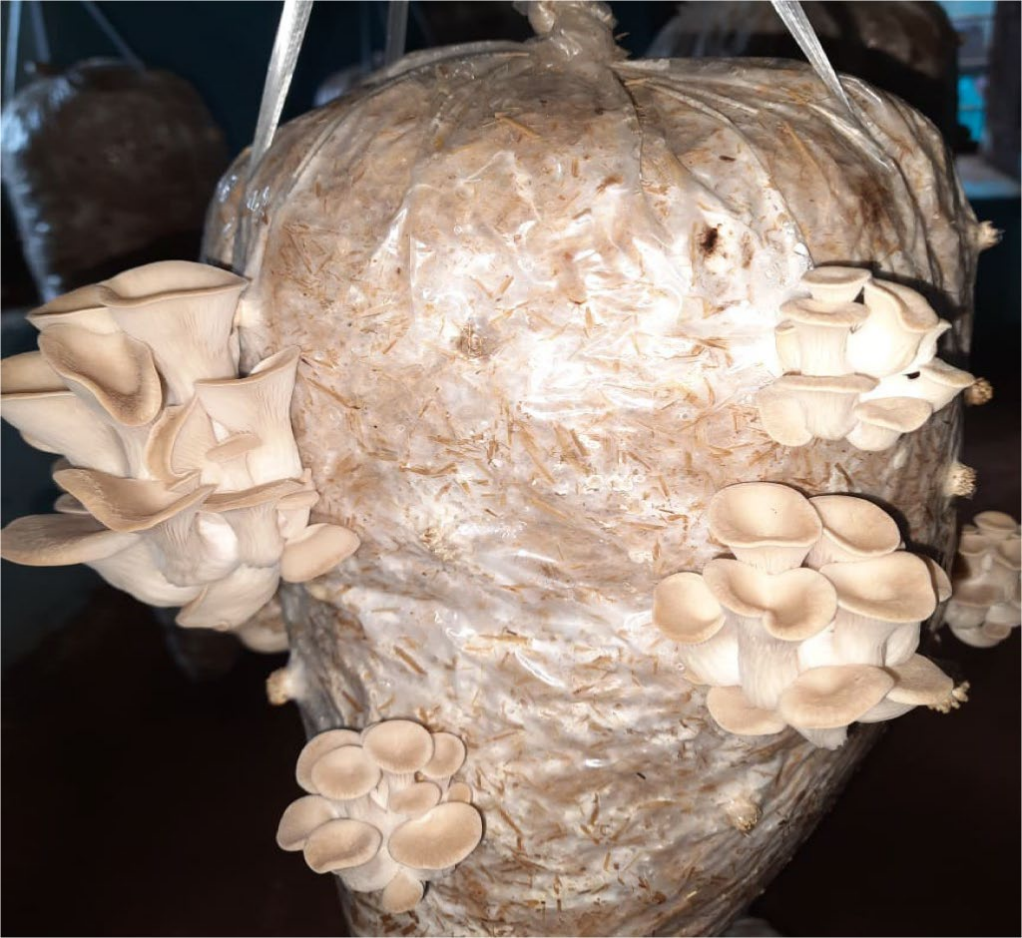
*Pleurotus ostreatus* cultivated (growing on substratum).

**Figure 3:**
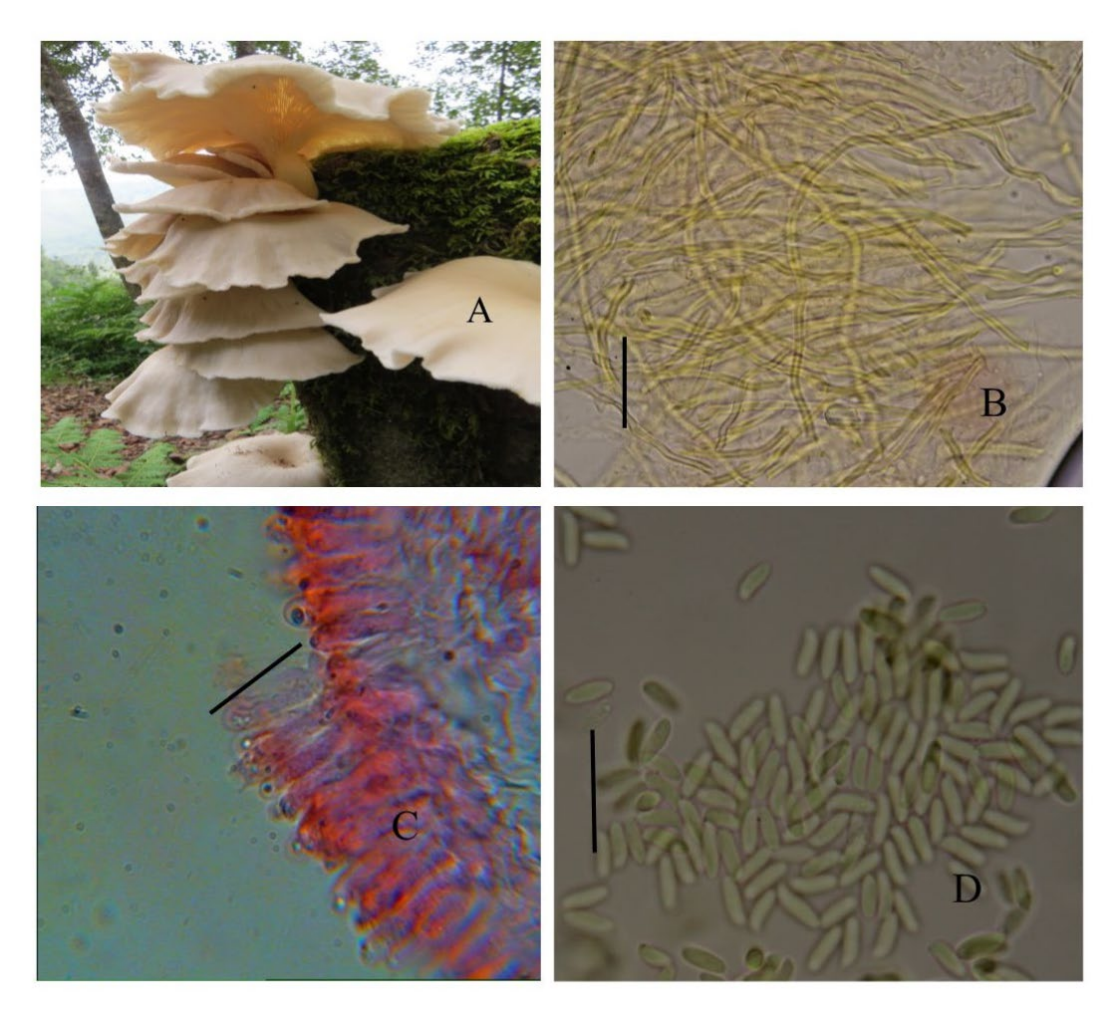
*Pleurotus ostreatus* wild (PoW)—(A) Basidiomata, (B) Transverse section of hymenium, (C) Contextual hyphae, (D) Basidiospores.

The cultivated variety (PoC) was already identified on the basis of its morphological features (Figure 2). The wild variety (PoW) was identified on the basis of macro and microscopic characters (Figure 3).

### 3.2 Antibacterial activity of BAPs

**Figure 4:**
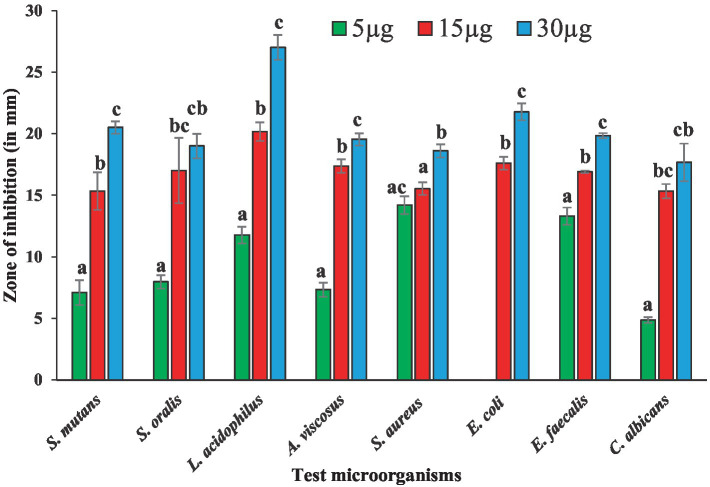
Graph showing antibacterial activities of BAPs from *P. ostreatus* cultivated against selected dental caries pathogens.

**Figure 5:**
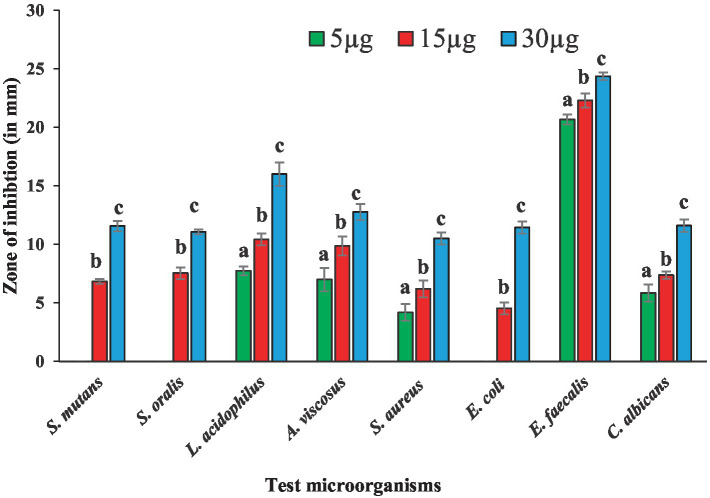
Graph showing antibacterial activities of BAPs from *P. ostreatus* wild against selected dental caries pathogens.

Antibacterial susceptibility of the tested bacterial pathogens showed that the *S. aureus* was resistant to methicillin (5.0 μg/disc),. The fungal pathogen *C. albicans* was resistant to nystatin (50 μg/disc). Antimicrobial activities of BAPs from PoC and PoW, represented in form of ZOIs, have been shown in Table 2 and Figures 4, 5. On an average, the ZOIs produced by the BAPs of PoC were larger than the ZOIs produced by BAPs of PoW, except for *E. coli*. Both PoC and PoW also showed maximum and minimum activity against different bacterial strains. The PoC showed the maximum and minimum ZOIs against *L. acidophilus* (20.16 ± 0.76 mm) and *S. aureus* (15.53 ± 0.50 mm), respectively at 15 μg/well concentration. At 30 μg/well concentration PoC showed the maximum and minimum ZOIs against *L. acidophilus* (27.0 ± 1.0 mm) and *S. aureus* (18.6 ± 0.52 mm), respectively. On the other hand PoW showed maximum ZOIs against *E. faecalis* (22.3 ± 0.60 mm and 24.36 ± 0.32 mm) at 15 μg/well and 30 μg/well concentrations, respectively. The PoW showed minimum ZOIs against and *E. coli* (4.53 ± 0.50 mm) and *S. aureus* (10.5 ± 0.50 mm), at 15 μg/well and 30 μg/well concentrations, respectively. The difference between antimicrobial activity of the BAPs from PoC and PoW was found significant after *t*-test analysis, at *p* < 0.05.

**Table 2:** Comparison of antibacterial activities (zone of inhibitions) of BAPs from *P. ostreatus* cultivated and *P. ostreatus* wild with tested antibiotics.

Microorganism	BAPs from PoC			BAPs from PoW			Amoxycillin	Ampicillin	Methicillin	Azithromycin	Erythromycin	Clindamycin	Nystatin
	5 μg	15 μg	30 μg	5 μg	15 μg	30 μg	30 μg	10 μg	5 μg	30 μg	15 μg	10 μg	50 μg
*S. mutans*	7.1 ± 1.0	15.33 ± 1.5	20.5 ± 0.5	0	6.83 ± 0.20	11.56 ± 0.45	21.16 ± 0.15	16.26 ± 0.25	0	26.2 ± 0.2	21 ± 0	30.23 ± 0.25	#
*S. oralis*	7.96 ± 0.55	17 ± 2.6	19 ± 1.0	0	7.53 ± 0.50	11.06 ± 0.20	13.13 ± 0.11	8.16 ± 0.15	0	23.2 ± 0.2	23.2 ± 0.2	26.2 ± 0.17	#
*L. acidophilus*	11.76 ± 0.68	20.16 ± 0.76	27 ± 1.0	7.73 ± 0.37	10.43 ± 0.51	16 ± 1.0	30.16 ± 0.20	30.23 ± 0.25	0	24.13 ± 0.15	26.3 ± 0.26	29.2 ± 0.17	#
*A. viscosus*	7.33 ± 0.57	17.36 ± 0.55	19.53 ± 0.50	7 ± 1.0	9.86 ± 0.80	12.76 ± 0.68	11.23 ± 0.25	10.2 ± 0.2	0	21.23 ± 0.20	23.36 ± 0.35	26.23 ± 0.20	#
*S. aureus*	14.2 ± 0.72	15.53 ± 0.50	18.6 ± 0.52	4.2 ± 0.72	6.2 ± 0.72	10.5 ± 0.5	28.23 ± 0.20	32.4 ± 0.4	0	26.23 ± 0.20	29.16 ± 0.15	32.16 ± 0.20	#
*E. coli*	0	17.6 ± 0.52	21.76 ± 0.68	0	4.53 ± 0.50	11.43 ± 0.51	20.26 ± 0.25	15.3 ± 0.3	0	22.16 ± 0.20	18.13 ± 0.15	0	#
*E. faecalis*	13.3 ± 0.7	16.9 ± 0.1	19.83 ± 0.20	20.66 ± 0.41	22.3 ± 0.60	24.36 ± 0.32	32.33 ± 0.30	32.1 ± 0.1	0	26.03 ± 0.05	29.1 ± 0.17	10.33 ± 0.41	#
*C. albicans*	4.86 ± 0.23	15.33 ± 0.57	17.66 ± 1.52	5.83 ± 0.72	7.36 ± 0.32	11.6 ± 0.52	*	*	*	*	*	*	0

*Antimicrobial activity was not performed as the target pathogen was a fungus. ^#^Antibacterial activity was not performed because Nystatin is an antifungal agent.

The growth of nystatin-resistant *C. albicans* was effectively controlled by the BAPs from both mushrooms even at 5.0 μg/well concentration, which was 10 times lower than the used amount of nystatin (50 μg). Notable observations were the higher ZOIs produced by the BAPs than the ZOIs produced by some standard antibiotics. For example, ZOIs produced by 15 μg of the BAPs from PoC were higher than the ZOIs produced by 30 μg of amoxicillin against *S. oralis*, and *A. viscosus.* The ZOIs produced by 30 μg of the BAPs from PoC were higher than the ZOIs produced by 30 μg of azithromycin against *L. acidophilus*. Against *E. faecalis,* the 5.0 μg amount of the BAPs from PoC and PoW both, was more effective than the 10 μg of clindamycin.

### 3.3 MIC and MBC

The MIC and MBC values of both the BAPs against the eight microbial pathogens are listed in Table 3. The MIC values of both the BAPs were 2.0 μg/mL, except for *E. coli* and *E. faecalis* (with the BAPs of PoW). The MBC, for both the BAPs against all the microbial strains used, was obtained at 4.0 μg/mL endpoint, except for *E. coli* and *E. faecalis*, for which the MBC was reported as 16 μg/mL with BAPs of PoW. Unlike the ZOIs values, the MIC and MBC values for the BAPs from PoC and PoW were similar against all the strains used in the study, except for *E. coli* and *E. faecalis.*

**Table 3:** MIC and MBC values of BAPs from *P. ostreatus* cultivated and *P. ostreatus* wild against the microorganisms.

Microorganisms	MBC/MIC (μg/mL)	
	PoC	PoW
*S. mutans*	4/2	4/2
*S. oralis*	4/2	4/2
*L. acidophilus*	4/2	4/2
*A. viscosus*	4/2	4/2
*S. aureus*	4/2	4/2
*E. coli*	4/2	16/8
*E. faecalis*	4/2	16/8
*C. albicans*	4/2	4/2

### 3.4 Time–kill study

**Figure 6:**
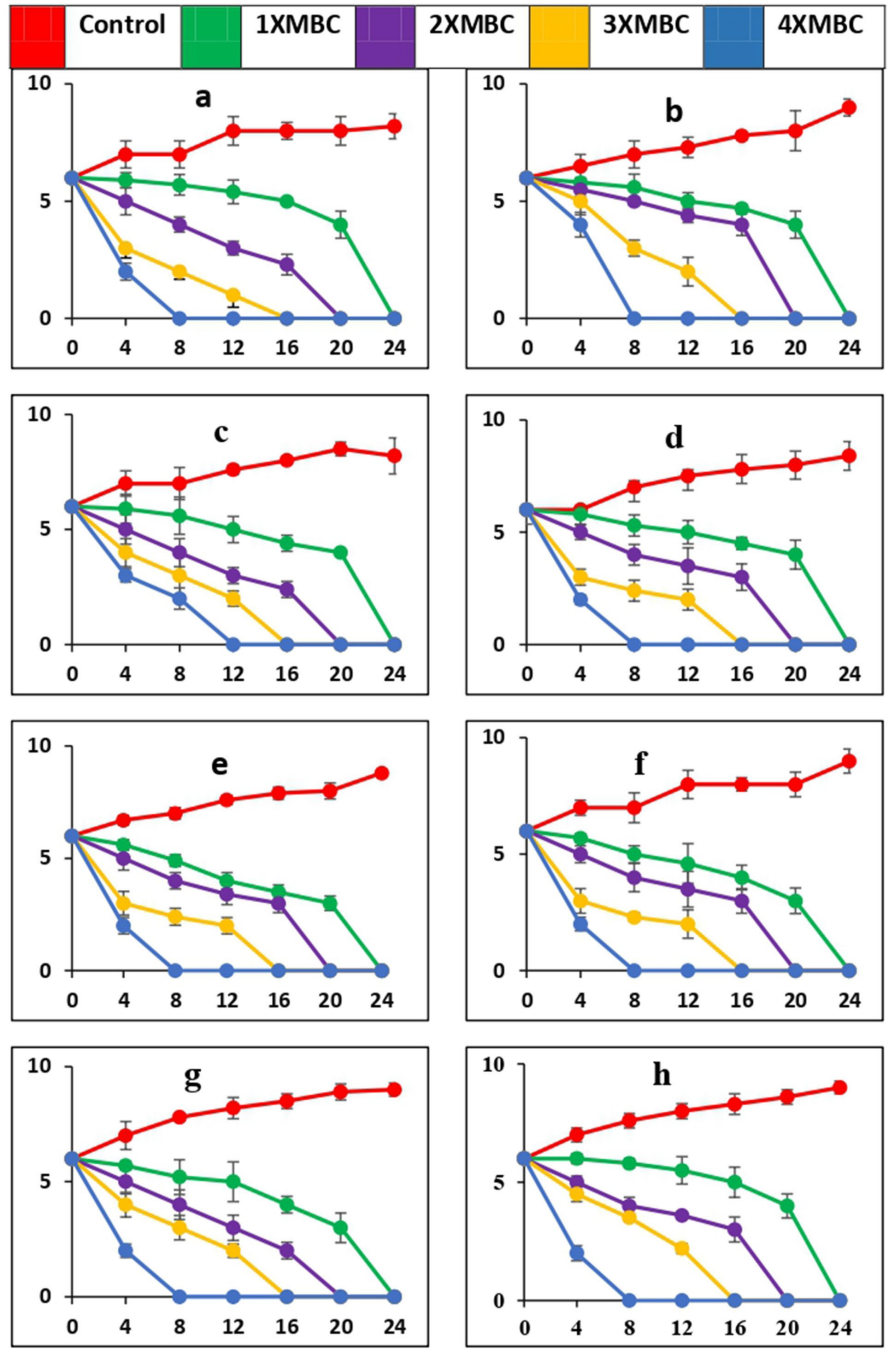
Time-kill curves of antimicrobial peptides of *P. ostreatus* cultivated against pathogens (a = *S. mutans*, b = *S. oralis*, c = *L. acidophilus*, d = *A. viscosus*, e = *S. aureus*, f = *E. coli*, g = *E. faecalis*, h = *C. albicans*). The pathogens were cultivated in a medium that included BAPs of PoC at concentrations equivalent to 1-, 2-, 3-, and 4-fold of the MBC, as specified for each curve in every plot. The nomenclature of the microbial species is provided in the y-axis caption in all of the eight graphs. Please take note of the logarithmic scale used for the y-axis representing colony-forming units (CFU). The microorganisms that remained viable were cultured and distributed onto agar plates at different intervals, as indicated on the x-axis. The data points in this study indicate the mean value plus or minus the standard deviation of three separate studies, each with a sample size of three.

**Figure 7:**
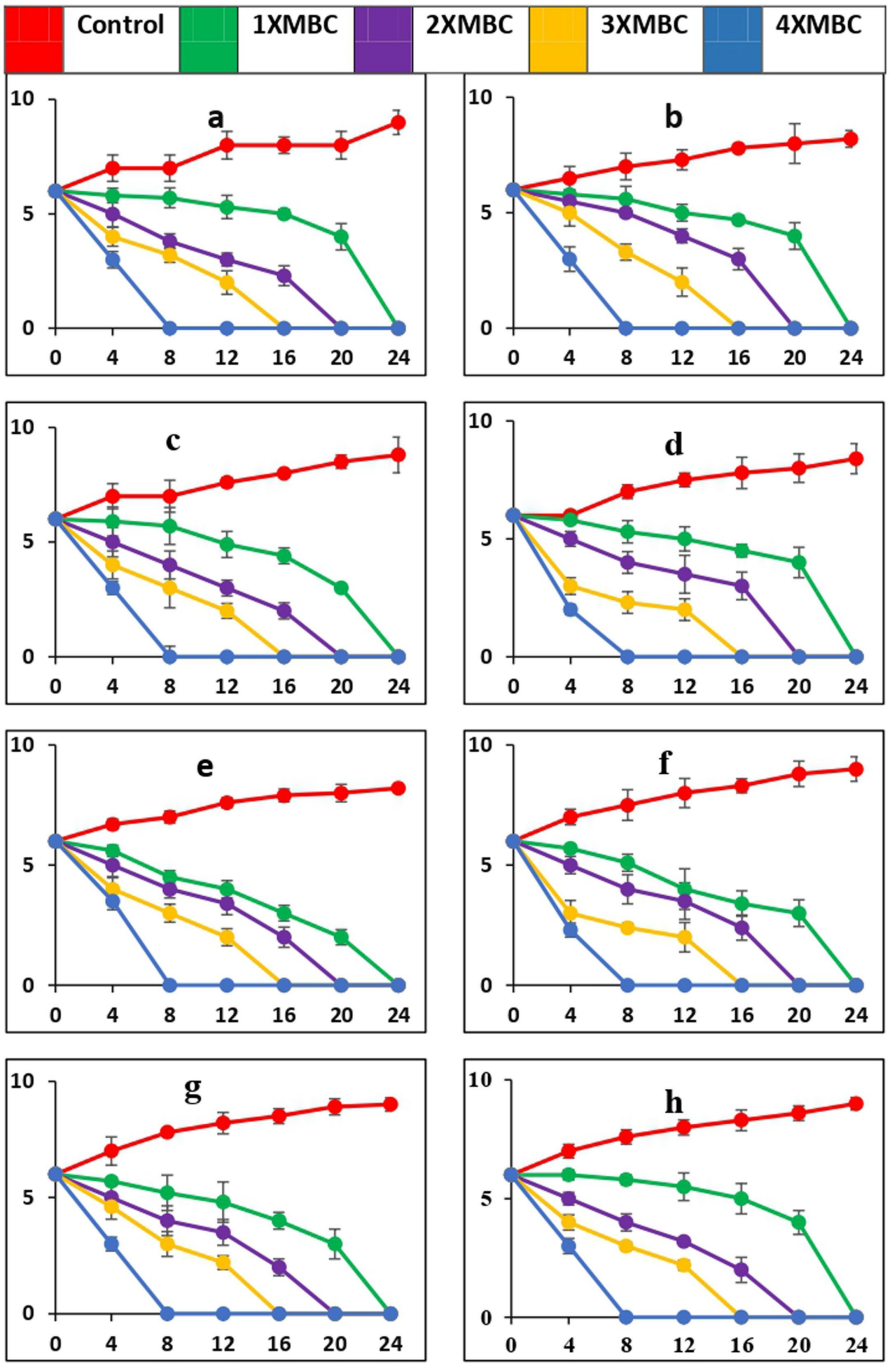
Time-kill curves of BAPs of *P. ostreatus* wild against pathogens (a = *S. mutans*, b = *S. oralis*, c = *L. acidophilus*, d = *A. viscosus*, e = *S. aureus*, f = *E. coli*, g = *E. faecalis*, h = *C. albicans*). The microorganisms were cultivated in a growth medium supplemented with BAPs derived from PoW at concentrations that were 1-, 2-, 3-, and 4-fold higher than the MBC, as specified for each curve in every graph. The nomenclature of the microbial species is provided in the y-axis caption in all of the eight graphs. Please take note of the logarithmic scale used for the y-axis representing colony-forming units (CFU). The microorganisms that remained viable were cultured and distributed onto agar plates at different time intervals, as indicated on the x-axis. The data points in this study represent the mean ± standard deviation (SD) of three independent trials, with a sample size (n) of 3.

The time-kill curves, against all the microbial pathogens used in this study, with BAPs from PoC and PoW is presented in Figures 6a–h, 7a–h, respectively. The time-kill results with BAPs from PoC (Figure 6) indicate that after 4 h of incubation, the order of difficulty to eradicate the bacteria was: *S. oralis* > *L. acidophilus* ≥ *C. albicans ≥ E. faecalis = S. mutans* = *A. viscosus = S. aureus = E. coli* (on the basis of number of colonies appeared on agar plates, CFU data not shown). The time-kill results with BAPs of PoW (Figure 7) were different than that of PoC, and indicate that after 4 h of incubation, the order of difficulty to eradicate the bacteria by PoW was: *S. aureus* ≥ *S. oralis* ≥ *C. albicans ≥ L. acidophilus* = *E. faecalis = S. mutans* ≥ *A. viscosus = E. coli* (on the basis of number of colonies appeared on agar plates, CFU data not shown). The time-kill results for BAPs from PoC (Figure 6) indicated that *S. oralis* and *L. acidophilus* survived for the longest time in the presence of BAPs obtained from PoC, followed by *C. albicans* and *E. faecalis*. For BAPs from PoW, *S. aureus* and *S. oralis* survived for the longest period followed by *C. albicans* and *L. acidophilus*.

While PoC exhibited a killing speed as fast as PoW at 4-fold of MBC, the absolute value of concentration was much high for PoW (4xMBC = 64 μg/mL, for *E. coli* and *E. faecalis*) than PoC (4xMBC = 16 μg/mL for *E. coli* and *E. faecalis*). The cidal potential of BAPs from both the mushrooms against remaining microbial strains was similar. After 4 h a log 2-fold to 4-fold reduction was observed and after 8 h most of the microorganisms were completely eradicated at 4X MBC of BAPs of both mushrooms.

### 3.5 Determination of biofilm inhibitory activity of BAPs from selected mushrooms

**Figure 8:**
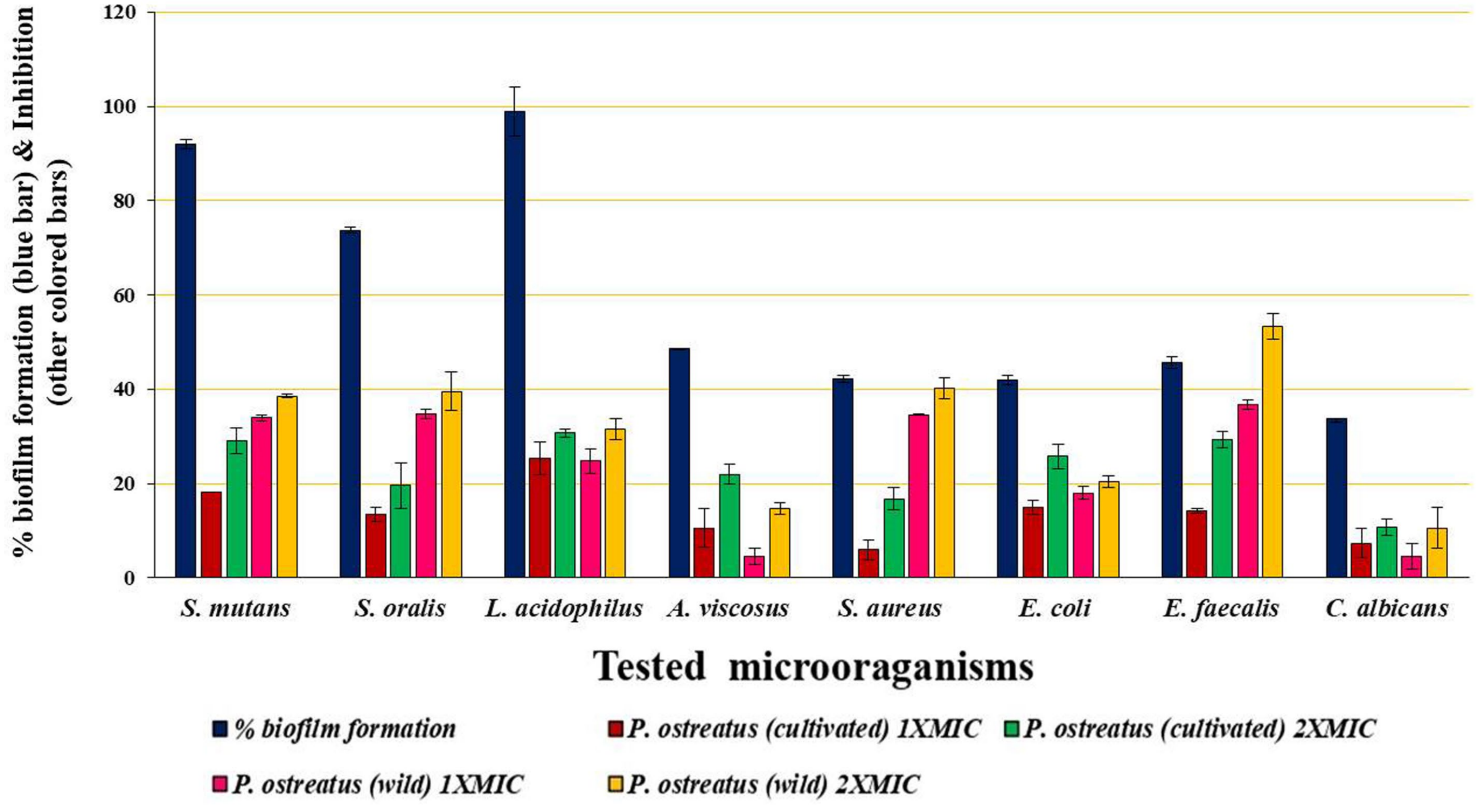
Graph showing biofilm formation (without BAPs) and biofilm inhibition (with BAPs) at 1X and 2X MIC concentrations.

The term “biofilm inhibition” refers the inhibition of the attachment and colonization of the microbial cells on a surface by the BAPs. All the microbial pathogens tested were biofilm producers, and the results of biofilm inhibition (in %) by the BAPs form PoC and PoW have been shown in Table 4 and Figure 8.

**Table 4:** Biofilm formation (in %) without BAPs and biofilm inhibition (in %) by BAPs of PoC and PoW at 1X and 2X MIC concentrations.

Microorganisms	Biofilm formation (%)	Biofilm inhibition by the BAPs (%)			
		With 1X MIC		With 2X MIC	
		PoC	PoW	PoC	PoW
*S. mutans*	91.9 ± 1.0	18.21 ± 0.06	33.96 ± 0.60	29.07 ± 2.64	38.58 ± 0.33
*S. oralis*	73.73 ± 0.57	13.44 ± 1.47	34.71 ± 0.98	19.54 ± 4.88	39.55 ± 1.08
*L. acidophilus*	98.9 ± 5.29	25.28 ± 3.4	24.73 ± 2.48	30.71 ± 0.79	31.55 ± 2.22
*A. viscosus*	48.56 ± 0.11	10.57 ± 4.08	4.48 ± 1.73	21.97 ± 2.14	14.68 ± 1.13
*S. aureus*	42.16 ± 0.68	5.91 ± 2.17	34.55 ± 0.08	16.70 ± 2.37	40.14 ± 2.24
*E. coli*	41.9 ± 1.0	15.01 ± 1.52	17.94 ± 1.37	25.71 ± 2.69	20.40 ± 1.28
*E. faecalis*	45.7 ± 1.21	14.18 ± 0.56	36.69 ± 0.97	29.27 ± 1.66	53.33 ± 2.75
*C. albicans*	33.8 ± 0.7	7.36 ± 3.06	4.63 ± 2.72	10.81 ± 1.77	10.56 ± 4.37

Maximum biofilm formation showed by *L. acidophilus* (98.9 ± 5.29%), followed by *S. mutans* (91.9 ± 1.0%) and *S. oralis* (73.73 ± 0.57%). All these three bacteria are involved in the initiation and progression of the dental caries, and *S. mutans* infection is one of the most prominent causes of dental caries. BAPs from PoC and PoW effectively inhibited the biofilm formation of these three and other microbial pathogens under observation. The biofilm inhibitory effect of the BAPs was dose-dependent. Biofilm of *E. faecalis* causing persistent apical periodontitis was most negatively affected by the BAPs of PoW at both 1X and 2X MIC concentrations, underlining their importance in persistent periodontitis. Biofilms of *A. viscosus*, *S. aureus,* and *C. albicans* were among the least affected (Table 4).

Comparison of the biofilm inhibition potential showed that for both 1X and 2X MIC values, the BAPs from PoW were more potent biofilm inhibitors than the BAPs of PoC, against all the bacterial biofilms tested in the study, except for *L. acidophilus* for which the BAPs from both the mushrooms showed similar biofilm inhibitory potential.

### 3.6 Antioxidant assays

#### 3.6.1 DPPH assay

The free radical scavenging activity of the different concentrations (0.2, 0.4, 0.6, 0.8, and 1.0 mg/mL) of BAPs from the mushrooms under study was compared with ascorbic acid (Table 5). The reported DPPH scavenging potentials of the BAPs from PoC and PoW were (29.40, 38.83, 58.43, 66.96, and 78.76%) and (37.02, 50.09, 61.70, 72.41, and 88.38%) respectively, for 0.2–1.0 mg/mL concentration range. The lowest IC_50_ value was observed for the standard antioxidant, i.e., ascorbic acid (154.01 ± 15.7 μg/mL), while the IC_50_ values for the BAPs from PoC and PoW were 529.18 ± 10.34 μg/mL and 401.14 ± 63.83 μg/mL, respectively.

**Table 5:** Percentage scavenging of DPPH by PoC, PoW, and ascorbic acid.

Concentration	PoC	PoW	Ascorbic acid
0.2 mg/mL	29.40%	37.02%	57.46%
0.4 mg/mL	38.83%	50.09%	62.32%
0.6 mg/mL	58.43%	61.70%	68.57%
0.8 mg/mL	66.96%	72.41%	75.15%
1.0 mg/mL	78.76%	88.38 %	91.26%

The % inhibition and IC-50 value data indicated that BAPs from PoW were better antioxidant agents than PoC.

#### 3.6.2 Antihemolytic assay

The concentration-dependent assay was employed to determine the anti-hemolytic activity of the BAPs within a range of 100–1,000 μg/mL. The absorbance values of BAPs derived from samples treated with PoC ranged from 0.62 to 1.12, which is comparable to the absorbance values obtained from samples treated with L-ascorbic acid, ranging from 0.67 to 1.45. BAPs from PoC and PoW showed almost similar absorbance, although the absorbance values with PoC were slightly low, compared to that of PoW at lower concentrations (100–5,000 μg/mL). The results showed that at 500 μg/mL concentration BAPs from PoC and BAPs from PoW showed 57.45 and 60.67% inhibition of hemolytic activity, respectively, while the inhibition caused by L-ascorbic acid at the same concentration was 78.47%. In all cases, lysis of erythrocytes declined with the increased concentrations of BAPs and L-ascorbic acid. The findings are concisely presented in Table 6.

**Table 6:** Antihemolytic activity of BAPs form *P. ostreatus* cultivated, *P. ostreatus* wild, and L-ascorbic acid.

Samples	Absorbance at 540 nm of different samples						% inhibition of hemolysis (at 500 μg/mL)
	Positive control	Negative control	100 μg/mL	250 μg/mL	500 μg/mL	1,000 μg/mL	
PoC	1.45 ± 0.002	0.11 ± 0.004	0.62 ± 0.01	0.72 ± 0.01	0.88 ± 0.01	1.12 ± 0.01	57.45
PoW	1.45 ± 0.002	0.11 ± 0.004	0.68 ± 0.01	0.73 ± 0.01	0.92 ± 0.01	1.12 ± 0.01	60.67
L-ascorbic acid	1.45 ± 0.002	0.11 ± 0.004	0.67 ± 0.01	0.79 ± 0.05	1.16 ± 0.01	1.45 ± 0.01	78.47

## 4 Discussion

The buccal cavity supports a diverse microbial community comprising at least hundreds of microbial species (Paster et al., 2001). For evaluating the antimicrobial activity of BAPs extracted from the wild and cultivated varieties of *P. ostreatus* mushroom, eight representative species of oral pathogens were chosen. The justification of selecting these pathogens has been given in the introduction section.

Antimicrobial peptides (AMPs) are recognized for their ability to directly impact a diverse spectrum of microorganisms, including bacteria, yeast, and viruses. AMPs also exhibit other biological activities such as antioxidant, wound healing and immunomodulatory effects. These multifaceted characteristics of AMPs position them as promising alternatives to traditional antibiotics, especially in light of the significant resistance that pathogenic bacteria have developed against conventional antibiotic treatments. AMPs have been identified in a wide range of protein sources. In this context, it is anticipated that the proteins extracted from both wild and cultivated strains of *P. ostreatus* may also contain AMPs. To confirm the presence of AMPs in these proteins, a preliminary screenings of ‘Bioactive Protein Extracts’ (BAPs) were conducted, with the objective of showing the presence of AMPs in them. This screening would pave the way for future investigations aiming the isolation and purification of novel AMPs in pure form from these and other mushrooms.

The mechanism of action of antimicrobial peptides is diverse and appears to be different from that of conventional antibiotics (Chen and Jiang, 2023; Somase et al., 2024). The activity of BAPs against the methicillin resistant *S. aureus* (MRSA) used in the study supports this hypothesis. Methicillin, developed to treat pencillin resistant *S. aureus*, acts by targeting penicillin binding proteins 2a (PBP2a) thus interferes with cell wall strength and integrity (Abebe and Birhanu, 2023). Other beta lactam antibiotics (Amoxicillin and Ampicillin) also act upon PBPs (Gambo et al., 2023; Kapoor et al., 2017), but resistance against methicillin is developed due to mutation in mecA gene, which is different from the beta-lactamase dependent resistance. Inhibition of the MRSA by the BAPs of PoC and PoW, indicates that BAPs must have their targets different than PBPs and mecA gene. BAPs also inhibited the growth of nystatin resistant *C. albicans* (Maghoomi, 2023; Zhang et al., 2023), indicating non-ergosterol targets of the BAPs. The effectiveness of AMPs predominantly depends upon their structural properties, i.e., numbers, composition and charges on the amino acids involved. Since, the BAPs is a mixture of many AMPs thus growth inhibition of methicillin and nystatin resistant microorganism can be accounted for the presence of more than one types of AMPs in the BAPs of mushrooms. Thus initial screening of the BAPs can be useful for identifying more than one type of AMPs in the given source.

BAPs from both sources demonstrated good antimicrobial activity against all the dental caries pathogens in the study, at 15 and 30 μg/well concentration. The antimicrobial potential of the BAPs from PoC was superior to that of PoW, except for *E. faecalis*, at all the concentrations used. In our previous study, focused on the BAPs from *A. bisporus*, the ZOIs ranging from 11 to 19 mm against the bacterial pathogens, common in both the studies, were observed (Gangwar and Maurya, 2022). BAPs from the wild mushrooms such as *Mycena pura, Ganoderma resinaceum, Russula fragilis*, and *Inocybe grammata* showed growth inhibition of *E. coli, C. albicans*, and other nosocomial pathogens due to the presence of antibacterial proteins in these wild mushrooms (Hearst et al., 2010). Oli et al., (Oli et al., 2020) extracted BAPs from *Auricularia auricula-judae* mushroom using the protein extraction different from our study. They used acetone method of protein extraction and evaluated good antimicrobial potential of BAPs against *S. aureus, E. coli, C. albicans*, and other microbial pathogens of humans. The present study revealed that the BAPs were less effective than the standard antibiotics, (except against a few pathogens as mentioned in the result section, and evident from the ZOIs data given in Table 2) in their present form. This underlines the need of further purification and characterization of the pure peptides (AMPs) from these BAPs.

Unlike the agar plate results showing superior antimicrobial activity of PoC over PoW, the MBC/MIC values for BAPs from both mushroom varieties were comparable, except in the instances of *E. faecalis* and *E. coli*. For these two bacteria the MBC/MIC values of PoW were four-fold higher than the MBC/MIC values of PoC. The ratio of MBC/MIC value indicated that the BAPs were microbicidal in nature. Ethanolic extract of 19 wild edible mushrooms (other than PoC and PoW) showed MIC range from 0.625–5 mg/mL against *S. aureus, M. lueteus, E. coli, P. aeruginosa* and *K. pneumoniae*. The range of MBC against the same bacteria was 2.5–5 mg/mL, and MBC/MIC ratio varied from 1 to 4 mg/mL (Nowacka et al., 2014). The MIC value of BAPs reported in our study (2 μg/mL) was lesser than the MIC value of BAPs from *A. auricula-judae* (2.5–5 μg) (Oli et al., 2020).

Time-kill determines how fast an antimicrobial material can kill microorganisms. In our study all the pathogens were killed after 4 h at 4X MIC of the BAPs, which was better than the result of Oli et al. (2020) which took 8 h to kill the pathogens used. The CFU data, recorded after 4 h of incubation indicated that PoC was better than PoW, because PoC reduced the microbial count of six pathogens upto 4 folds, while PoW was able to reduce the microbial count of only two bacteria up to 4-fold within the same time. Moreover, the MBC value of PoW was much higher than PoC, indicating a two-fold higher antibacterial potential of the PoC compared to PoW (although the ZOIs values do not indicate the same fold change in antimicrobial potential of both). This could be due to differences in the nature of anti-microbial peptides present in BAPs. Due to the larger size of anti-microbial peptides, the rate of diffusion would be slower in agar plates resulting in lesser antimicrobial potential, while in solution (during MBC and MIC estimation) the movement of peptides was unrestricted. The time kill kinetics studies on the BAPs were unavailable, except for the Oli et al. (2020). The methanol extract of four Ghanaian mushrooms (*Trametes gibbosa, Trametes elegans, Schizophyllum commune,* and *Volvariella volvacea*) eradicated *E. coli*, *P. aeruginosa*, *K. pneumoniae*, *S. typhi*, *S. pyogenes S. aureus*, *E. faecalis*, *B. subtilis*, and *C. albicans* within 6–52 h. the MIC values of the used extract by them was much higher (4–20 mg/mL) than the MIC of BAPs used in present work (Appiah et al., 2017).

Three bacteria namely *L. acidophilus, S. mutans*, and *S. oralis* are responsible for the dental caries. In addition, *E. faecalis* is also associated with persistent apical periodontitis. Biofilms formed by these bacteria were effectively inhibited by the BAPs of both mushrooms. Inhibition of the biofilms formed by *L. acidophilus, S. mutans*, and *S. oralis* indicates that BAPs from the test mushrooms could prevent the initiation of dental caries, while inhibition of *E. faecalis* biofilm by BAPs indicates their importance in controlling the persistent cases of apical periodontitis. Unike our study, the antibiofilm potential of the metabolites present in the aqueous extracts but no the proteins of *Lentinus edodes*, *Macrolepiota procera*, *Armillaria mellea*, *P. ostreatus*, and *A. auricula-judae* and *Laetiporus sulphurous* was evaluated and 47.72–70.87% inhibition of the *S. aurues* biofilm was observed (Cuvalova et al., 2018; Papetti et al., 2018). These works (Oli et al., 2020; Nowacka et al., 2014) evaluated the antibiofilm efficacy of mushroom metabolites, but our work is focused on the antimicrobial proteins (BAPs) extracted from mushrooms, whose extraction protocol and chemical nature are entirely different from the metabolites. Our previous report on BAPs from *A. bisporus* also showed 18.82–67.52% inhibition of biofilms formed from different microbial pathogens including *S. aureus, S. mutans, S. oralis, L. acidophilus* etc. (Gangwar and Maurya, 2022). Interestingly, the BAPs from PoW showed better biofilm inhibition activity compared to the BAPs from PoC, which was contradictory to the ZOIs-based antibacterial potential. This again indicates the different mechanisms of actions of the BAPs from both the mushrooms. Our study also suggests the use of a combination of BAPs from both PoC and PoW to control dental caries problems, because, the biofilms composed of *S. mutans, S. oralis, S. aureus*, and *E. faecalis* were less affected by the BAPs of PoC, but were inhibited effectively by the BAPs of PoW. On the other hand, the biofilms composed of *A. viscosus, E. coli*, and *C. albicans* which were less affected by the BAPs of PoW, were effectively inhibited by the BAPs of PoC. Thus BAPs from both PoC and PoW complemented each other (Table 3).

Antimicrobial peptides represent a pivotal class of natural compounds renowned for their effectiveness as anti-biofilm agents (Yong et al., 2019). Extracted from various sources like frog skin (e.g., Japonicin-2LF and Dermaseptin-PT9), honey bee venom (Melittin), and red pepper (Capsicumicine), purified AMPs have showcased robust anti-biofilm properties. Unlike chemical antibiofilm agents, AMPs acts as anti-biofilm agents against both Gram-positive and Gram-negative bacteria. AMPs exhibit multifaceted methods of inhibiting biofilms, one of which involves influencing bacterial cell signaling. For instance, human Cathelicidin LL-37 has been observed to significantly diminish the quorum sensing (QS) system in *P. aeruginosa* at concentrations as low as 0.5 μg/mL, thereby impeding its biofilm formation (Di Somma et al., 2020). During biofilm initiation, quorum sensing inducers interact with membrane proteins like OmpA, fibronectin binding proteins, protein A, SasG, and other biofilm-associated proteins to produce QS molecules (Asma et al., 2022). AMPs intervene by interacting with bacterial membranes, disrupting the autoinducers-based QS signaling crucial for biofilm formation. The diverse chemical composition of QS signaling molecules and the array of molecular mechanisms involved in QS hint at the limitations of chemical QS inhibitors, which typically target either Gram-positive or Gram-negative bacteria exclusively. However, our study has reported the remarkable inhibition of biofilms formed by both Gram-positive and Gram-negative bacteria, showcasing the broad-spectrum efficacy of AMPs in combating biofilm formation across diverse bacterial types.

Besides antimicrobial and anti-biofilm activities, BAPs also showed potential antioxidant activities and were effective in protecting the erythrocytes from H_2_O_2_-induced hemolysis. Although low level of endogenous reactive oxygen species (ROS) is necessary for vital physiological activities, but the high level of ROS causes oxidative stress. If not addressed properly this oxidative stress could cause various diseases like diabetes, atherosclerosis and cancer. In food industry, ROS caused lipid peroxidation is also an important concern to improve quality and shelf life of the food items. AMPs also act as natural antioxidants, attracting the attention of researchers and the food industry for the development and maintenance of food product quality and safety through their addition (Tadesse and Emire, 2020). AMPs having 2–20 amino acids and rich in hydrophobic amino acids, such as proline, valine, tryptophan and phenylalanine, showed potent antioxidant activity (Ketnawa et al., 2018; Yang et al., 2019). In present work the BAPs also showed good antioxidant activity due to presence of AMPs in them.

During hemolysis the ROS primarily target hemoglobins and polyunsaturated fatty acids (PUFA) within erythrocytes. Consequently, these species lead to the degradation of erythrocyte membrane lipids and proteins, causing hemolysis and mutilation. Hemolysis occurs at a higher rate when erythrocytes are exposed to toxins like hydrogen peroxide (Karim et al., 2020). In this experiment, the BAPs from both mushrooms showed good antihemolytic potential and prevented erythrocyte membrane from H_2_O_2_-induced oxidative damage. According to the results, both the BAPs exhibited almost similar anti-hemolytic activity, but their hemolysis-preventing potential was ~23–27% less than L-ascorbic acid. DPPH-based antioxidant potential and antihemolytic activity of the mushrooms (other than BAPs) have been evaluated by different groups and wild mushrooms are reported to have antioxidant properties (Gangwar et al., 2023; Madhanraj et al., 2019; Sharif et al., 2017). The antioxidant and antihemolytic potential of the BAPs from cultivated *A. bisporus* has also been evaluated in our perevious study (Gangwar and Maurya, 2022). The potential mechanisms through which BAPs might prevent hemolysis involve AMPs within the BAPs forming a surface coating on erythrocytes. This coating may be able to restore membrane integrity damaged by high temperatures and exposure to hydrogen peroxide. Unlike the antibacterial potential indicated by the ZOIs the antihemolytic potential of the BAPs from PoW was slightly better than the BAPs of PoC, but the difference was statistically insignificant. This again confirms the general concept that wild varieties have good antioxidant potential than their cultavated counterparts.

### 4.1 Plausible mechanism of action of BAPs based on the experimental data

The BAPs under observation showed antimicrobial, antibiofilm and antioxidant activities. The cidal nature of BAPs (based on MIC/MBC ratio) indicates that rather than inhibiting the synthesis of protein/enzyme/metabolites, BAPs must target cell integrity by attacking bacterial cell envelope. AMPs are already know to target cell membrane by various mechanism hence cell membrane could be a probable target of BAPs. In addition, inhibition of the methicillin and nystatin resistant microbial pathogens indicates that BAPs are having intracellular targets other than the cell membrane. These intracellular targets must be different from PBPs in case of bacteria, and other than ergosterol in case of fungal pathogens, because two of the microbes (*S. aureus* and *C. albicans*) used in the study were having these target based resistance, and were inhibited by BAPs.

Biofilm involves different steps, i.e., attachment, formation and maturation of biofilms. The experimental data revealed that BAPs inhibited the initial attachment of the microbial cell and the growth of microbial biofilms. AMPs present in the BAPs are known to interact and bind the QS molecules, thus inhibition biofilm growth. Moreover, the antimicrobial nature of AMPs would also killed the persister cells of biofilm thus inhibiting further growth and spread of biofilms.

## 5 Conclusion

The study aimed to screen the presence of AMPs in the BAPs derived from PoC and PoW, and to compare their therapeutic potentials. Various assays, including agar well diffusion, MIC, MBC, time-kill kinetics, antibiofilm, antioxidant, and anti-hemolytic assays, were conducted to evaluate the antimicrobial and antioxidant properties of the BAPs.

The results indicated that both PoC and PoW complement each other across different antibacterial assays, providing comprehensive antimicrobial efficacy. In terms of the zone of inhibition values, PoC performed better, while PoW exhibited superior results in the antibiofilm assay, suggesting stronger inhibition of quorum-sensing molecules by the AMPs in PoW. The MIC, MBC, and time-kill kinetics showed that both PoC and PoW were nearly equal in efficacy, except against *E. coli* and *E. faecalis*. Regarding antioxidant potential, the wild variety PoW outperformed PoC, likely due to its natural habitat. However, the anti-hemolytic potential of both PoC and PoW was comparable. Thus, it can be concluded that although both PoC and PoW share common AMPs, as revealed by their comparable antimicrobial properties, they also contain distinct AMPs that account for the differences in their performance across various antimicrobial and antioxidant assays. Combining BAPs from both sources could provide a more effective antimicrobial and antioxidant solution.

Incorporating these versatile compounds into oral care products holds great potential for combating diverse microbial species responsible for dental caries. However, since PoW is a wild variety, its availability could become a limiting factor in large-scale production. In such cases, using PoC as the primary component and supplementing it with a limited amount of PoW could offer a sustainable solution.

## Data Availability

The raw data supporting the conclusions of this article will be made available by the authors, without undue reservation.
